# Supplementary material for: In Vitro Antiviral Activity of Hyperbranched Poly-L-Lysine Modified by L-Arginine against Different SARS-CoV-2 Variants
Source: Nanomaterials (Basel). 2023 Dec 6;13(24):3090. doi: 10.3390/nano13243090 (PMC10745586; doi:10.3390/nano13243090)
Supplement: Supplementary file 1 [file nanomaterials-13-03090-s001.zip › nanomaterials-2739587-supplementary.pdf]

nanomaterials

MDPI

Supplementary Materials

In Vitro Antiviral Activity of Hyperbranched Poly-L-lysine Modified by L-Arginine against Different SARS-CoV-2 Variants

Federico Fiori [1], Franca Lucia Cossu [1], Federica Salis [1], Davide Carboni [1], Luigi Stagi [1], Davide De Forni [2], Barbara Poddesu [2], Luca Malfatti [1], Abbas Khalel [3], Andrea Salis [4], Maria Francesca Casula [5], Roberto Anedda [6], Franco Lori [2] and Plinio Innocenzi [1,*]

1 Laboratory of Materials Science and Nanotechnology (LMNT), CR-INSTM, Department of Biomedical Sciences, University of Sassari, Viale San Pietro 43/B, 07100 Sassari, Italy; federico.fiori@studenti.unipg.it (F.F.); cossufrancalucia@libero.it (F.L.C.); f.salis3@studenti.uniss.it (F.S.); dcarboni@uniss.it (D.C.); lstagi@uniss.it (L.S.); luca.malfatti@uniss.it (L.M.)
2 ViroStatics srl, Viale Umberto I 46, 07100 Sassari, Italy; d.deforni@virostatics.com (D.D.F.); b.poddesu@virostatics.com (B.P.); f.lori@virostatics.com (F.L.)
3 Department of Chemistry, College of Science, United Arab Emirates University, Al Ain P.O. Box 15551, United Arab Emirates; abbask@uaeu.ac.ae
4 Department of Chemical and Geolocial Sciences, University of Cagliari, Cittadella Universitaria SS 554 Bivio Sestu, 09042 Monserrato, Italy; asalis@unica.it
5 Department of Mechanical, Chemical and Materials Engineering, University of Cagliari, Via Marengo 2, 09123 Cagliari, Italy; mariaf.casula@unica.it
6 Porto Conte Ricerche srl, Strada Provinciale S.P. 55, Loc. Tramariglio, 07041 Alghero, Italy; anedda@portocontericerche.it
* Correspondence: plinio@uniss.it

Table S1. pH of the precursor and products solutions. The concentration used for the measurements is 1 mg mL^{-1} for each sample.

Sample (1 mg mL^{-1})	pH
**L-lysine**	10.0 (6.8 + 3.2)
**L-arginine**	10.2 (6.8 + 3.4)
**HBPL** – (Lys:BA = 1:1)	8.5 (6.8 + 1.7)
**LBA0.1** – (Lys:BA:Arg = 1:1:0.1)	9.5 (6.8 + 2.7)
**LBA0.25** – (Lys:BA:Arg = 1:1:0.25)	8.6 (6.8 + 1.8)
**LBA0.5** – (Lys:BA:Arg = 1:1:0.5)	8.9 (6.8 + 2.1)
**LBA1**-(Lys:BA:Arg = 1:1:1)	8.3 (6.8 + 1.5)
**LBA2** – (Lys:BA:Arg = 1:1:2)	7.2 (6.8 + 0.4)
**BAA** – (Arg:BA = 1:1)	7.9 (6.8 + 1.1)

Table S2. Hydrodinamic diameter (size), %of intensity, Standard deviation for main peak and ◎-Potential of the synthesized nanopolymers.

Sample	Hydrodinamic diameter (nm)	% of Intensity	Standard deviation for main peak (nm)	Zeta-Potential (mV)
HBPL	233.1	96.1	60.13	20.0 ± 2.0
LBA0.1	178.6	99.2	83.97	23.5 ± 4.0
LBA0.2<br>5	466.1	95.2	280.5	10.4 ± 4.1
LBA0.5	293.4	99.0	112.3	22.9 ± 3.5
LBA1	270.5	94.0	167.1	20.3 ± 3.5
LBA2	1269	95.3	326.3	7.8 ± 2.8
BAA	353.1	92.6	164.0	10.1 ± 4.5

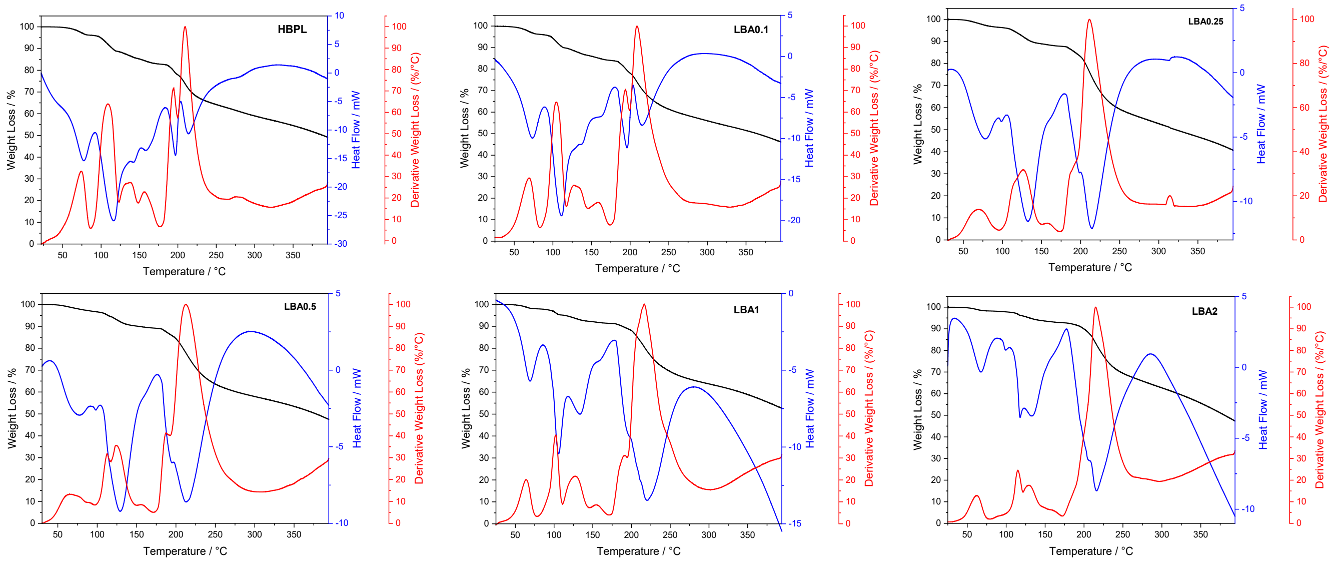

Figure S1. TGA-DSC analysis of the samples with increasing amounts of L-arginine. The sample of L-lysine catalyzed by boric acid (HBPL) is reported as reference.

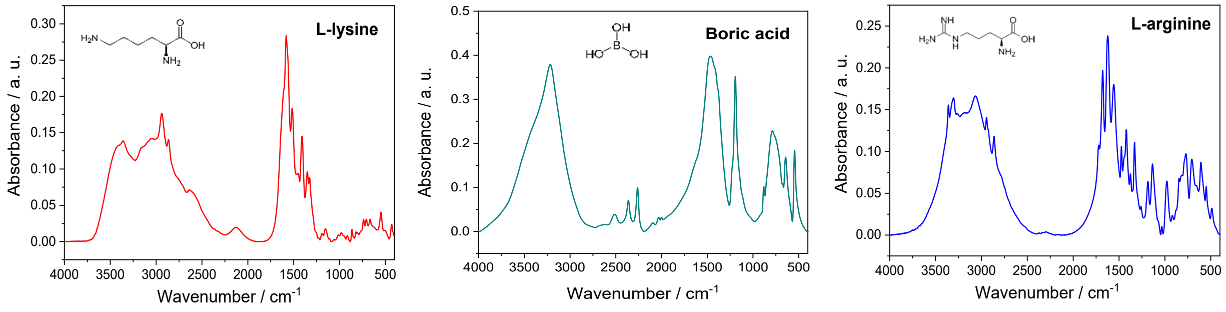

Figure S2. FTIR absorption spectra of L-lysine, boric acid and L-arginine.

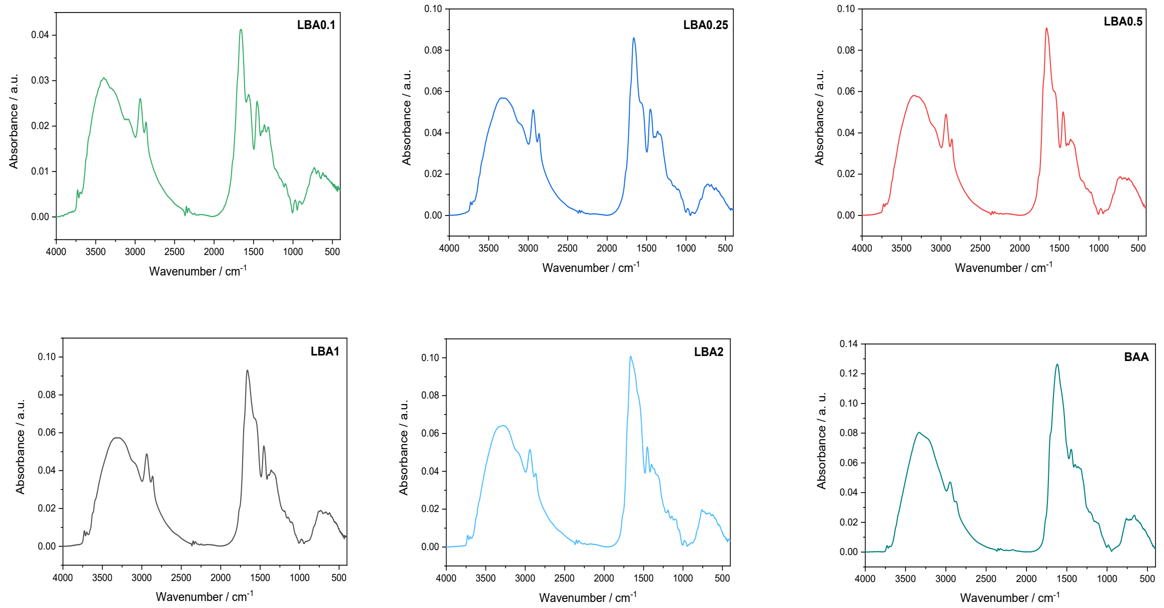

Figure S3. FTIR absorption spectra of the samples prepared at increasing amounts of L-arginine.

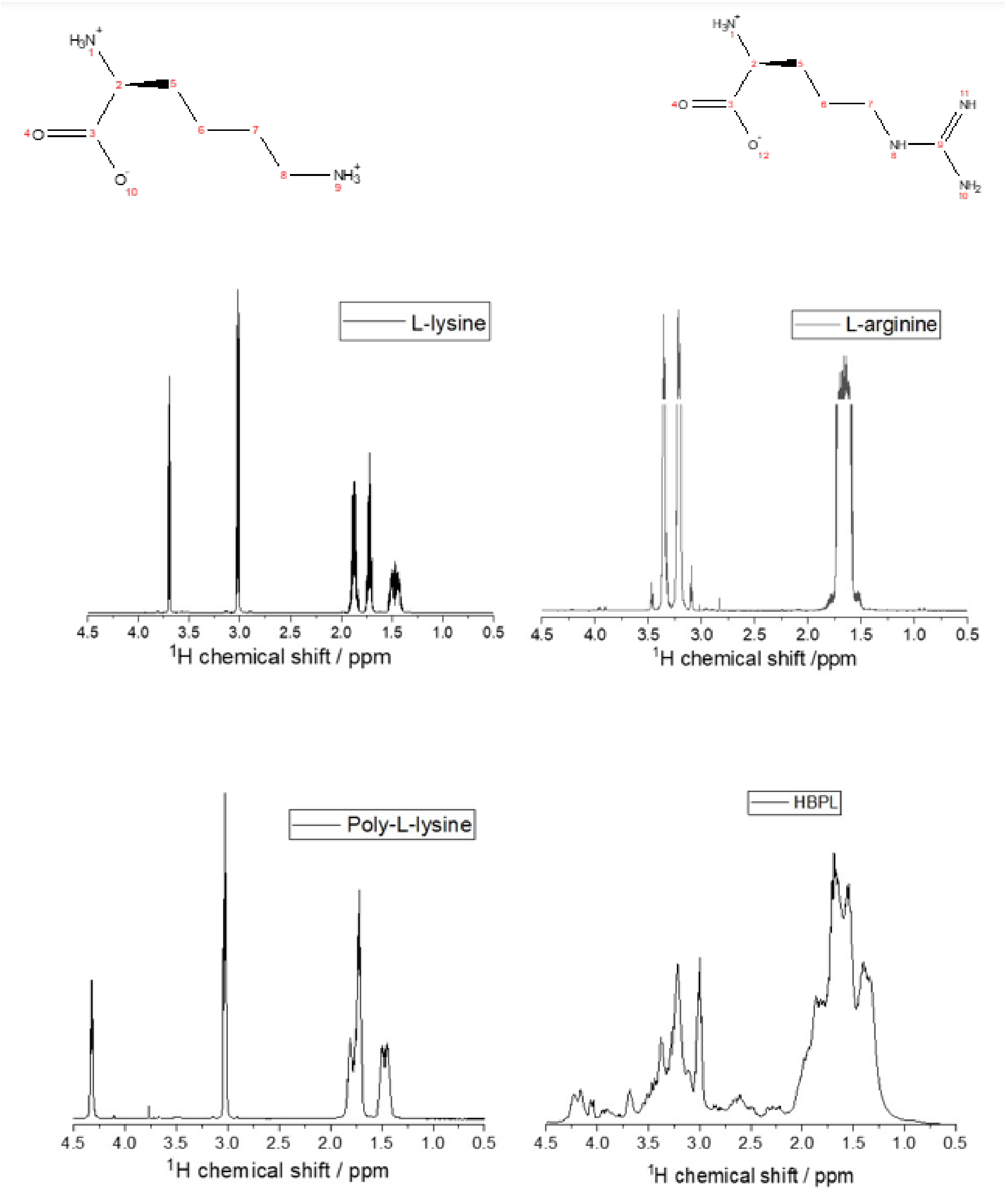

Figure S4. (Top) ^{1}H NMR reference spectra of L-lysine and L-arginine. (Bottom) ^{1}H NMR reference spectra of Poly-L-lysine (Sigma-Aldrich) and Hyperbranched Poly-L-lysine (HBPL).

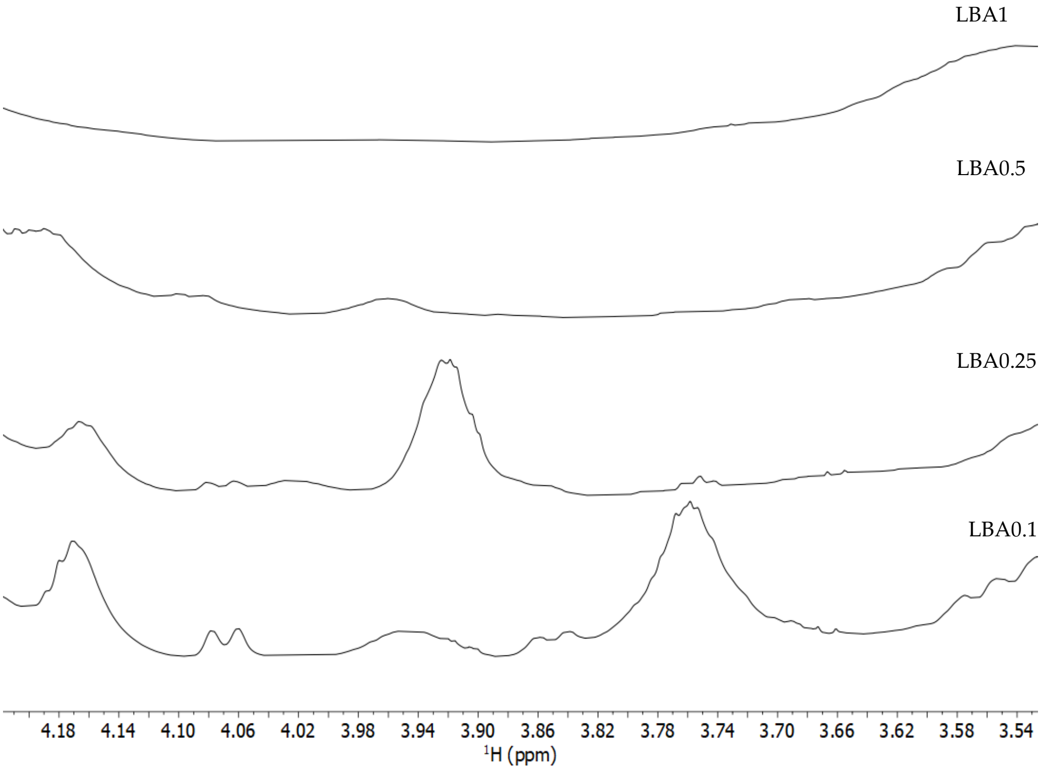

Figure S5. Expanded 3.5-4.2 ppm region of the 1D ^{1}H NMR spectra as a function of increasing L-arginine in the series LBA0.1, LBA0.25, LBA0.5 and LBA1.

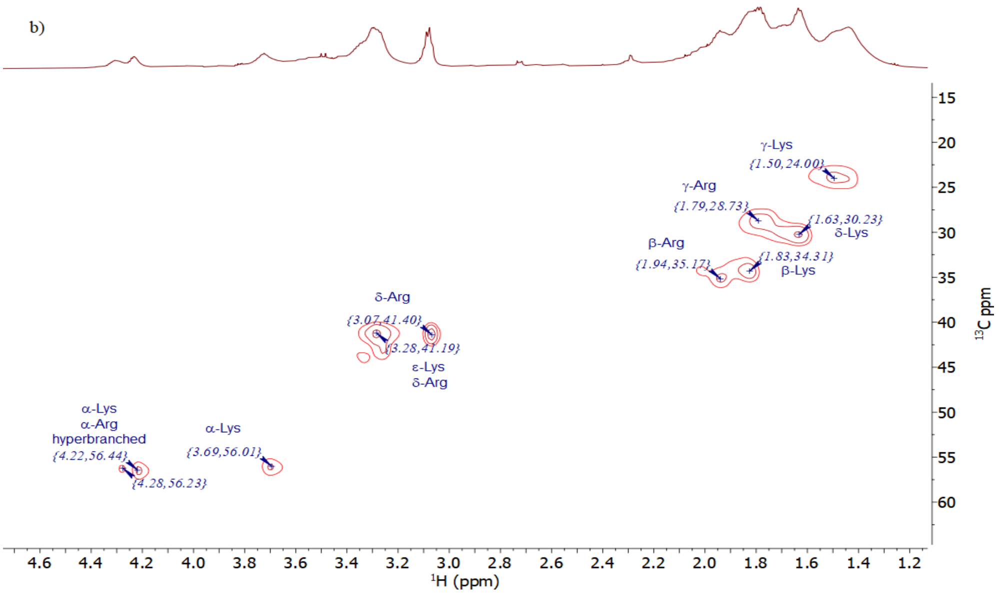

Figure S6. Two-dimensional NMR spectra of LBA0.1 showing heteronuclear (^{1}H-^{13}C HSQC) correlations.

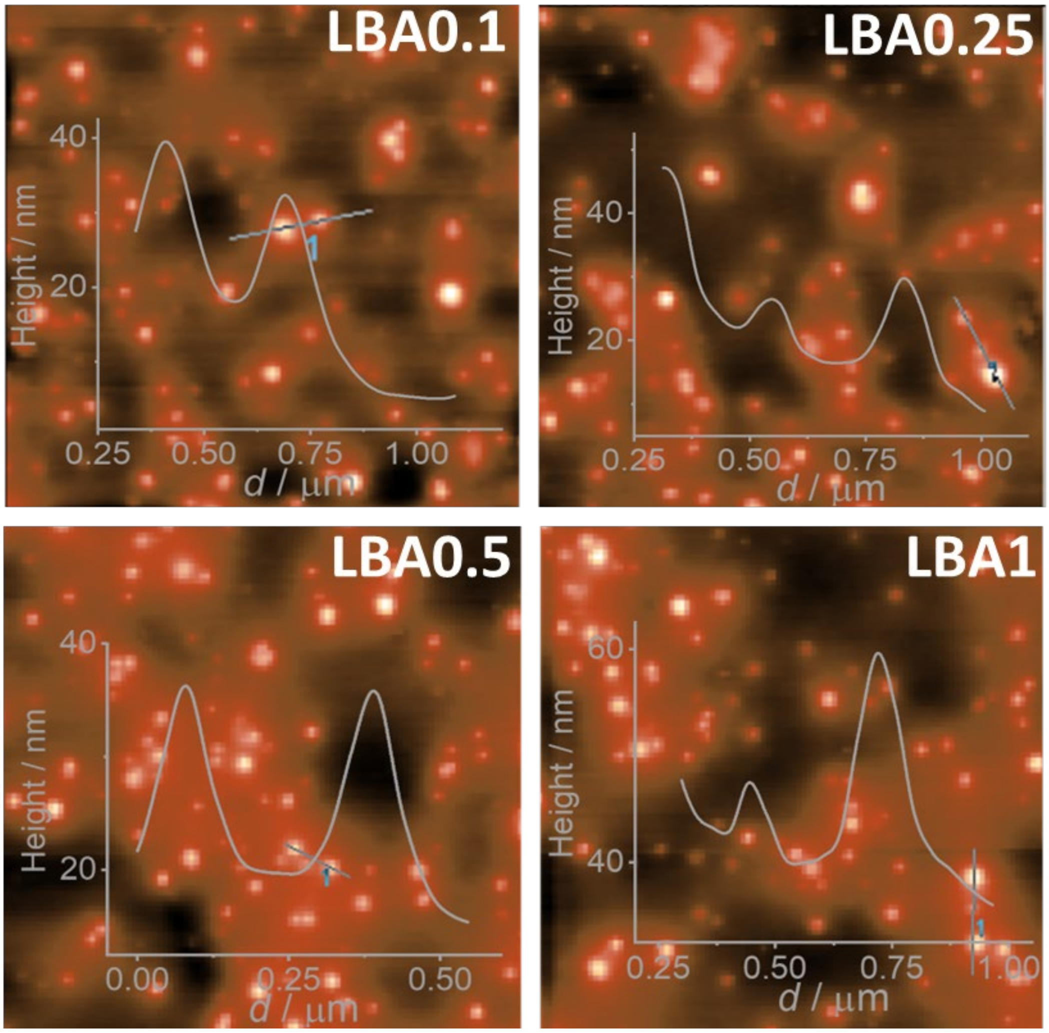

Figure S7. AFM measurement in tapping mode of the nanopolymers at different L-lysine(Lys)/Boric acid (BA)/L-arginine(Arg) ratio. Lys:BA:Arg = 1:1:0.1 (LBA0.1); Lys:BA:Arg = 1:1:0.25 (LBA0.25); Lys:BA:Arg = 1:1:0.5 (LBA0.5); Lys:BA:Arg = 1:1:1 (LBA1).

Table S3. Cytotoxicity evaluated using MTS assay for LBA1, LBA0.5, LBA0.25, LBA0.1 and BAA. HBPL and Remdesivir have been used as reference. The data are reported as a function of the concentration (in ⊚g mL^{-1}) with the mean and standard deviation obtained in the MTS assay.

Sample	Conc. (μg mL^{-1})	Mean	St. dev.	Cytotoxic Concentration 50% (μg mL^{-1})
LBA1	100	24.2	3.2	25
	20	57.3	1.9	
	4	84.1	10.2	
	0.8	94.0	2.3	
LBA0.5	100	47.0	8,5	100
	20	72.7	12,5	
	4	61.2	7,3	
	0.8	80.5	16,6	
LBA0.25	500	58.2	0.3	>500
	100	99.5	0.6	
	20	104.0	6.7	
	4	104.0	2.3	
LBA0.1	500	54.7	0.6	>500
	100	77.2	4.4	
	20	100.4	0.6	
	4	100.1	4.5	
BAA	500	79.2	3.0	>500
	100	89.7	12.9	
	20	101.0	0.0	
	4	104.8	0.1	
HBPL	100	71.7	1.7	>100
	20	97.9	2.3	
	4	107.9	5.1	
	0.8	100.6	3.3	
Remdesivir	6	105.5	2.3	>10
	1.2	102.0	1.0	
	0.24	106.7	0.2	
	0.048	103.6	5.3	
